# THE ASSOCIATION OF URINARY INCONTINENCE AND DISABILITY AMONG A DIVERSE SAMPLE OF MID-LIFE SWAN WOMEN

**DOI:** 10.1093/geroni/igad104.2033

**Published:** 2023-12-21

**Authors:** Sheila Dugan, Sybil Crawford, Karla Wente, L Waetjen, Carrie Karvonen-Gutierrez, Sioban Harlow

**Affiliations:** Rush University System for Health, Chicago, Illinois, United States; U Mass Chan Medical School, Worcester, Massachusetts, United States; Rush University, Chicago, Illinois, United States; University of California, Davis, Sacramento, California, United States; University of Michigan, School of Public Health, Ann Arbor, Michigan, United States; University of Michigan, Ann Arbor, Michigan, United States

## Abstract

Few studies have examined the prospective association between urinary incontinence (UI) and disability in midlife women. We assessed associations between UI type, frequency and amount with reported disablity in a racially/ethnically diverse cohort of community dwelling midlife women. Data was from longitudinal analyses of questionnaires from the multi-center, prospective cohort Study of Women’s Health Across the Nation. We used multivariable ordinal logistic regression to examine if UI type, frequency and amount at the 13th follow-up were associated with the World Health Organization Disability Assessment Schedule (WHODAS) at the 15th follow-up (controlling for other factors, e.g. menopause status, body mass index, lifestyle and psychosocial factors) and disability at Follow-up 13. UI was associated with subsequent reports of disability in participants, particularly in the WHODAS domains of mobility, communication, and societal participation. Associations were strongest for mixed UI type compared to stress UI or urge UI, daily frequency of UI compared to monthly or weekly, and larger amounts of urine leakage compared to drops of leakage. UI appears had a strong association with multiple domains of disability in midlife women after two years of follow-up. It is important for clinicians to address UI earlier in symptom onset. Screening for mixed UI and UI that occurs more frequently and it larger amounts, more specifically, may yield better information regarding a patient’s future disability risk.

